# Sensor Selection to Improve Estimates of Particulate Matter Concentration from a Low-Cost Network

**DOI:** 10.3390/s18093008

**Published:** 2018-09-08

**Authors:** Sinan Sousan, Alyson Gray, Christopher Zuidema, Larissa Stebounova, Geb Thomas, Kirsten Koehler, Thomas Peters

**Affiliations:** 1 Department of Public Health, East Carolina University, Greenville, NC 27834, USA; 2 Department of Occupational and Environmental Health, University of Iowa, Iowa City, IA 52242, USA; alyson-gray@uiowa.edu (A.G.); larissa-stebounova@uiowa.edu (L.S.); thomas-m-peters@uiowa.edu (T.P.); 3 Department of Environmental Health and Engineering, Johns Hopkins Bloomberg School of Public Health, Baltimore, MD 21205, USA; czuidema@jhu.edu (C.Z.); kkoehle1@jhu.edu (K.K.); 4 Department of Industrial and Systems Engineering, University of Iowa, Iowa City, IA 52242, USA; geb-thomas@uiowa.edu

**Keywords:** PM, aerosol exposure, low-cost sensors, low-cost wireless network, occupational monitoring, sensor calibration, sensor selection

## Abstract

Deployment of low-cost sensors in the field is increasingly popular. However, each sensor requires on-site calibration to increase the accuracy of the measurements. We established a laboratory method, the Average Slope Method, to select sensors with similar response so that a single, on-site calibration for one sensor can be used for all other sensors. The laboratory method was performed with aerosolized salt. Based on linear regression, we calculated slopes for 100 particulate matter (PM) sensors, and 50% of the PM sensors fell within ±14% of the average slope. We then compared our Average Slope Method with an Individual Slope Method and concluded that our first method balanced convenience and precision for our application. Laboratory selection was tested in the field, where we deployed 40 PM sensors inside a heavy-manufacturing site at spatially optimal locations and performed a field calibration to calculate a slope for three PM sensors with a reference instrument at one location. The average slope was applied to all PM sensors for mass concentration calculations. The calculated percent differences in the field were similar to the laboratory results. Therefore, we established a method that reduces the time and cost associated with calibration of low-cost sensors in the field.

## 1. Introduction

The Occupational Safety and Health Administration (OSHA) requires that a worker’s exposure to respirable particulate matter (PM), those particles that can penetrate to the alveolar regions of the lungs [1], is less than 5 mg/m^3^ in an 8-h, time-weighted, average concentration. For regulatory purposes, respirable particulate matter is measured with a gravimetric filter or federal equivalent methods [2,3]. However, high-accuracy, gravimetric methods are expensive and only provide time-weighted average measurements [4]. Increasing the number of filter measurements spatially and temporally allows exploration of exposure variability to establish appropriate control methods. However, in practice, few filter measurements are actually collected because increasing the number of measurements will increase costs considerably. In addition, the regulatory framework discourages additional sampling due to the increasing probability of measuring an exceedance of the occupational exposure limits [5]. 

Direct-reading instruments can provide rapid information on particle concentrations in the workplace. The Personal Dust Monitor (PDM 3700, Thermo Scientific., Waltham, MA, USA) uses a filter for continuous measurement of PM with a tapered element oscillating microbalance [6]. The PDM 3700 concentration measurements were evaluated for different aerosols and were found to have a near 1:1 linear relationship compared to gravimetric measurements [7]. Light-scattering instruments, such as photometers (e.g., personal DataRAM 1500, pDR-1500, ThermoFisher Scientific., Waltham, MA, USA) measure the light scattered from an assembly of particles at a fixed angle. The scattered light increases linearly with particle mass concentration [8]. To use a photometer to estimate particle concentration for a particular aerosol, the photometer output must be scaled and offset to match filter-based measurements because light scattering depends on the refractive index and size distribution of particles. Although providing excellent temporal resolution, direct-reading instruments are expensive (photometers, >$6000; PDM3700, $17,000), limiting the number that can be purchased and consequently restricting information on spatial variability. 

Low-cost sensors based on light scattering have proved useful in both environmental [9,10,11] and occupational studies [12,13]. One low-cost optical particle counter, the DC1700 ($400, Dylos Corporation, Riverside, CA, USA) [14], was used in multiple indoor [15], environmental [9], and occupational [12] exposure studies. Another low-cost original equipment manufacturer photometer, the Sharp GP ($12, GP2Y1010AU0F) [16], has also proved useful after appropriate calibration. For an indoor exposure study, Patel, et al. [17] calibrated the Sharp GP against a Sidepak AM510 photometer (TSI Inc., Shoreview, MN, USA), achieving an *R*^2^ of 0.71.

The low-cost monitors have enabled researchers to develop air quality monitoring systems that involve networks of tens or scores of devices. Bhattacharya, et al. [18] developed such a system to measure carbon monoxide (CO) and carbon dioxide (CO_2_) gases, PM, temperature, and relative humidity that transmitted and stored the measurements in a remote database. Kim, et al. [19] developed a low-cost indoor air quality wireless network that measures CO, volatile organic carbon, and PM in residential buildings in order to control indoor climate systems. Rajasegarar, et al. [20] developed a network of wireless sensors to measure CO, CO_2_, nitrogen oxide, methane, PM concentrations, and temperature, both indoors and outdoors. Zikova, et al. [21] deployed an outdoor network of sensors to measure spatial and temporal PM variability in a metropolitan area.

Relatively few researchers have emphasized calibration strategies to refine the accuracy and precision of low-cost sensor networks, a problem presenting unique challenges because each sensor needs individual calibration. Increasing accuracy and precision requires individual site calibration that is time-consuming and increases labor costs. Gao, Cao and Seto [11] deployed a network of low-cost PM sensors co-located with filter-based measurements at eight outdoor locations for one week in the city of Xi’an, China. Before deployment, linear calibration was established between seven sensors and filter-corrected measurements from a DustTrak II photometer (Model 8532, TSI, Shoreview, MN, USA), co-located at one site for four days. Schneider, et al. [22] deployed a network of 24 low-cost gas sensors at various kindergarten locations in Oslo, Norway. The authors first co-located the gas sensors with their counterpart reference instruments at one location to perform on-site linear calibration for three months to reduce measurement bias and error. For practical purposes and to gain sensor-specific calibration, both studies co-located and calibrated the low-cost sensors at one location before deployment to save effort and cost. These studies do not address calibration methods for a network of sensors in the field. Additional work should be performed to establish a basis for calibrating a network of low-cost sensors and resolving methods for reducing calibration time for all the sensors in the field. 

The objective of the current study was to establish a method to select low-cost sensors that respond similarly prior to their incorporation into a PM sensor network. First, we established a laboratory Average Slope Method to select 50% of the sensors that have a similar response from a group of 100 sensors when calibrated with a specific aerosol type and a reference instrument. The 50% selection is arbitrary and could be altered by the end user. Second, we evaluated our Average Slope Method with an Individual Slope Method to establish the feasibility of our proposed approach. Our sensor selection method was based on calculating and applying the mean slope to all the sensors for mass calculation. This method was compared with calculating mass using sensor-specific slope. Finally, we tested our laboratory selection method in the field by conducting a field calibration procedure and comparing sensor measurements in the field with reference instruments. This work is essential because co-locating a network of sensors at one location for field calibration may not be feasible. In addition, calibrating one sensor will save time and calibration for sensor replacement in the field.

## 2. Methods and Materials

### 2.1. Sensor Selection Methods and Rationale

#### 2.1.1. Method 1: Average Slope Method

The proposed method was to select a subset of PM sensors that had similar slopes in a laboratory experiment, allowing us to use a single slope determined in the field for all PM sensors in the sensor network [23]. The method selects 50% of the PM sensors within a Z percent criterion that were within ±20% around the average slope for all the sensors. If needed, the ±20% criterion can be adjusted to allow selection of more or fewer sensors. In the laboratory, a linear calibration was performed on a group of (*n*) random PM sensors and a reference instrument with a specific aerosol type. The linear regression takes the simple form

(1) $$\;Y_{i}=m_{i}X+b_{i}\;$$

where *Y_i_* represents the sensor output, *X* is the concentration from the reference instrument, and *m_i_* and *b_i_* are the slopes and intercepts for the calibrated sensors from *i* equal to 1 to *n*, respectively.

The sensor mass concentration ($MC_{i}$) for each PM sensor (*i*) was then calculated based on the linear regression model

(2) $$\;MC_{i}=\frac{Y_{i}-b_{i}}{m_{i}}\;$$

For Method 1 described above, we replaced m_i_ with the average slope ($\bar{m})$ value for the selected sensors:

(3) $$\;\bar{m}=\frac{1}{ns}\overset{ns}{\underset{i=1}{\sum }}m_{i}\;$$

where *ns* = the total number of selected sensors based on Method 1.

In contrast to the calculated mean slope, we used the intercepts (*b_i_*) calculated for each PM sensor from Equation (1). The calibration approach for slope and intercept is different. The slope is dependent on the aerosol type and therefore must be calculated on-site. In contrast, the intercept represents the sensor value measured at zero concentration (particle-free air). Therefore, laboratory calibration is sufficient for intercept calculation and application in the field.

#### 2.1.2. Method 2: Individual Slope Method

For the second method, the same group of sensors selected via Method 1 was evaluated; however, the individual slope for each PM sensor was used instead of the average slope for the chosen PM sensors. Similar to Method 1, we used the intercepts (*b_i_*) calculated for each PM sensor from Equation (1). Here, we compared the difference between applying an average slope versus a sensor-specific slope to calculate mass concentration from sensor output.

### 2.2. Laboratory Evaluation of PM Sensors

We used Sharp GP sensors as an example. The Sharp GP has a small form-factor (0.046 × 0.03 × 0.0176 m), is low cost (~$12), and has performed well under environmental and occupational settings [23]. The sensor operates in passive mode with an infrared diode for particle light scattering and a phototransistor that captures the intensity of the scattered light. The Sharp GP sensor, in addition to hazardous gas, noise, relative humidity, and temperature sensors, were operated with a microcomputer inside a sealed plastic case (0.2 × 0.1 × 0.11 m) in a custom air quality monitor. The microcomputer recorded measurements every 2 s and stored each sensor output locally and also broadcasted the data wirelessly to an on-site server. Validation of other sensors and description of the custom monitor are described in the literature; the gas sensors were evaluated by Afshar-Mohajer, et al. [24], the noise sensor was designed and evaluated by Hallett, et al. [25], and the custom monitors are described by Thomas, et al. [26].

**Figure 1:**
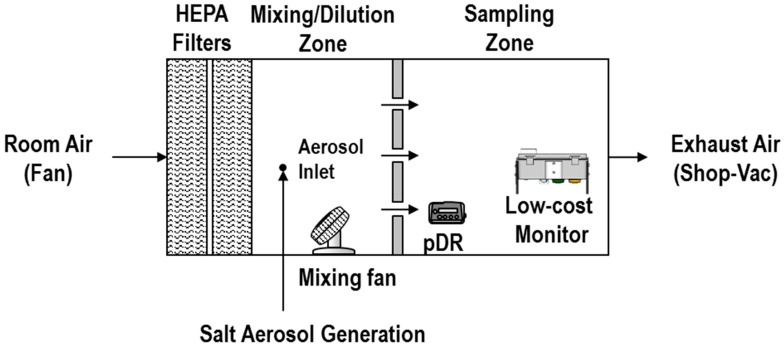
Experimental set up used to select the Sharp particulate matter (PM) sensors.

The experiments were conducted inside a laboratory chamber, as shown in Figure 1. The chamber consisted of a mixing zone (0.64 × 0.64 × 0.66 m) and a sampling zone (0.53 × 0.64 × 0.66 m) divided by a perforated plate positioned in the middle of the chamber. A monitor (not to scale) that contains the Sharp GP sensor with the other sensors and the microcomputer is shown in the sampling zone. A pDR-1500 operated with an inlet cyclone (cutoff diameter of 2.5 µm) was also positioned in the sampling zone. Clean air was supplied (0.25 m^3^/min) to the chamber using two HEPA filters and mixed with a small fan in the mixing zone. Salt is a common test aerosol and was used to conduct the calibration experiments. A vacuum, referred to as Shop-VAC, was used at the exit of the chamber to ensure proper ventilation of the chamber content. A salt solution (0.9% *w*/*v*, #7210, Fisher Scientific, Pittsburgh, PA, USA) was nebulized with a vibrating mesh (Aeroneb Solo System, Aerogen, Galway, Ireland) operated by a voltage regulator to control aerosol generation and achieve different concentrations. The salt particles were dried with silica gel before entering the chamber and then diluted by the clean air in the mixing zone. Size distribution information for the same salt solution has been published by the authors [23], where the size distribution was similar for different concentrations.

The chamber did not have enough space to accomodate 100 monitors in the sampling zone. Therefore, three experiments were conducted: two experiments included 36 monitors and one experiment included 34 monitors. We used three monitors in all three experiments to test the repeatability of the three experiments and confirm that the chamber homogeneity was similar in each experiment. Six different steady-state salt concentrations were generated, including zero air, for a period of 5 min (hereinafter referred to as the six-point concentration). At each steady state, the monitors and the pDR-1500 were set to record data every 2 s. The pDR-1500 dataset was not filter corrected for the laboratory experiment. The maximum target chamber concentration was 300 µg/m^3^, with four measurements targeted inbetween the zero air and maximum concentration. This maximum concentration was based on typical maximum concentrations observed inside a heavy-manufacturing facility as measured with pDR-1500 measurements (raw readings without filter correction). The filter correction factor was not nessasry for sensor selection since the correction factor would be applied to all sensors after field calibration.

For all three methods, we compared the calculated mass concentrations from the sensors with the pDR-1500 measurements using the Environmental Protection Agency (EPA) percent difference calculation (*d_i_*) [27]:

(4) $$\;di\;=\;\frac{A_{i}-C}{C}\;\times 100\;$$

where *A_i_* and *C* are the mass concentrations a for the PM sensors and pDR-1500, respectively. Excluding zero air, each sensor measured five different steady-state concentrations for 5 min. Therefore, each sensor had five 1-min averages represented by d values for each of the five steady-state concentrations, providing a total of 25 percent differences. However, the National Institute for Occupational Safety and Health (NIOSH) specifies that the percent difference for gas sensors should be within and not exceed ±10%. It is important to mention that NIOSH uses the term “bias” for Equation (4), which should not be confused with bias described below. EPA does not define a percent difference criterion for PM sensors. For percent differences, NIOSH guideline for gas sensors are used for PM sensors because it is the only guideline available.

Physical measurements are associated with fundamental noise that results in a random error and can be decreased by increasing the number of measurements and averaging times [28]. The standard error, or the percent difference in our case, can be reduced by increasing the averaging time for the same collected measurements. For paired measurements, increasing the number of measurements used to calculate the means decreases the error. For example, for 1000 paired measurements taken every 2 s, the error based on 5-min averages is lower than 1-min averages, and the error for 1-min averages is lower than 5-s averages. Therefore, increasing the number of measurements is crucial for increasing the averaging times and reducing the error.

To demonstrate the effectiveness of different averaging times, we compared the percent differences calculated in Method 1 that are based on *t* = 1-min averages with two other averaging times: *t* = 5-s averages and *t* = 5-min averages. Since the microcontroller records data every 2 s, two measurements were averaged at *t* = 5 s. The percent difference calculation in Equation (4) was performed using Ai and C averages based on 5-s, 1-min, and 5-min averages calculated from the 2-s data.

We evaluated the limit of detection (LOD) for our lowest concentration generated inside the chamber based on Zikova, et al. [29] method. The method was used to evaluate that the lowest concentrations are within the LOD of the continuous PM measurements for the sensors. The collocated measurements must be performed for nonzero concentrations and are considered as evidence at the 99% confidence level [30]. Zikova, Hopke and Ferro [29] estimated LOD for collocated PM sensors, with continuous measurements, which provide mean concentrations that exceed three times the standard deviation. Therefore, the ratio of mean to standard deviation was calculated for the selected collocated sensors based on Method 1, measuring the lowest concentration generated for each of the three experiments.

EPA has a Performance Evaluation Program for PM instruments that calculates bias [27] as

(5) $$B_{i}=\;\frac{1}{k}\sum_{i=1}^{k}\frac{A_{i}-C}{C}\times 100\;=\;\;\frac{1}{k}\sum_{i=1}^{k}d_{i}\times 100\;$$

where $B_{i}$ represents the mean percent difference for all the measurements taken by a single PM sensor (*i*). EPA specifies that the percent bias goal for acceptable measurement uncertainty should be within ±10%. In addition, k  represents the five steady-state concentrations based on 5-min averages.

Method 1 was used as the selection criterion and calibration for the bias calculation. The *A_i_* and *C* were based on 5-min averages calculated from the 2-s data for all the paired measurements at different concentrations. The 5-min averaging time was used to obtain five steady-state concentrations during a 25-min period. Therefore, each sensor has one bias value for all the measurements taken at different steady-state concentrations.

The confidence interval for the mean bias values for Equation (5) can be calculated as [27]

(6) $$\;Upper\;90\%\;Confidence\;Interval=B_{i}+t_{0.95,df}\frac{Sd_{i}}{\sqrt{k}}\;$$

(7) $$\;Lower\;90\%\;Confidence\;Interval=B_{i}-t_{0.95,df}\frac{Sd_{i}}{\sqrt{k}}\;$$

where $t_{0.95,df}$ is 95th quantile of a *t*-distribution and $Sd_{i}$ is the standard deviation of the percent differences.

### 2.3. Application in Field

Based on Method 1, we chose 50% of the 100 sensors calibrated in the laboratory that have a similar response. We then tested Method 1 in the field by deploying 80% of the chosen PM sensors at different locations in an indoor manufacturing site for a duration of two weeks. Twenty percent of the sensors selected were stored for backup in case one of the deployed sensors failed. The field test was conducted at a heavy-manufacturing facility. The area of the facility floor where measurements were made was large (75,000 m^2^). The manufacturing processes conducted on the floor of the facility included welding, cutting, and grinding at different locations. A total of 38 locations were selected. Three monitors were installed at 1 location, referred to as the supersite, and one monitor was installed at each of the remaining 37 locations. Each monitor broadcast measurements wirelessly to an on-site server. Measurements were saved to the server every 5 min. First, we calibrated three PM sensors with a reference instrument at the supersite, and we calculated the average slope from the three sensors. Then, the average slope was used to calculate the mass based on Equation (2) for all the PM sensors in the network, with the laboratory calculated intercepts for each sensor applied. We then compared the calculated mass with a reference instrument by collocated 1-min measurements with a reference instrument at each location. The 1-min collocation time was chosen to ensure that we could complete all measurements within 8 h.

A pDR-1000 (Thermo Scientific., Waltham, MN, USA) was deployed at the supersite location alongside the three monitors. The pDR-1000 was chosen because, similar to the PM sensors, it operates in passive mode without a pump. The pDR-1000 was set to record measurements every 5 min, which was equivalent to the 5-min measurements for the PM sensors stored on the server. The pDR-1000 mass concentration measurements were corrected using filter-based measurements. A 37-mm polytetrafluoroethylene (PTFE) membrane filter (R2PJ037, PALL, Port Washington, USA) was co-located with the pDR-1000 at the supersite for a duration of 4 h. The PTFE membrane filter was positioned inside a 37-mm filter cassette attached to an aluminum respirable dust cyclone (225-01-02, SKC, Eighty Four, PA, USA) operated with GilAir Plus pump (Sensidyne, St. Petersburg, FL, USA). The filter-corrected pDR-1500 mass concentrations, *C* corrected as follows

(8) $$\;C\;corrected\;at\;time\;t=unadjusted\;C\;at\;time\times \frac{\bar{G}measured}{\bar{C}measured}\;$$

where *C* represents the mass concentrations for the pDR-1500 recorded at *t* = 1 s, $\bar{G}measured$ is the mass concentration measured gravimetrically with the PFTE membrane filter internal to the cyclone, and $\bar{C}measured$ is the mean of unadjusted pDR-1500 mass concentrations measured over 4 h.

The average time-paired 5-min voltage (mV) measurements from the three PM sensors were calibrated with the filter-corrected pDR-1000 measurements. The linear regression slope was obtained and used for mass concentration calculation for all the PM sensors at all the locations. 

For field validation, a pDR-1500 was used at each of the monitor locations to perform 1-min collocated measurements. Readings from a monitor and the reference instrument were recorded simultaneously using a wired connection to a computer. The same pDR-1500 used in the six-point laboratory calibration experiment was used in the field validation. The pDR-1500 operated with an inlet cyclone (cutoff diameter of 4 µm) operating in active mode with a 37-mm glass microfiber filter (934-AH, Whatman, MN, USA) at the outlet. Mass concentrations for the PM sensors were calculated using the average slope obtained from the supersite and the individual intercepts calculated from Equation (1) in the six-point laboratory experiments. The pDR-1500 measurements were corrected using the 37-mm glass microfiber filter. The filter-corrected pDR-1500 mass concentrations were calculated from Equation (8), where *C* represents the mass concentrations for pDR-1500 recorded at *t* = 1 s, $\bar{G}measured$ is the mass concentration measured gravimetrically with the glass microfiber filter internal to the pDR-1500, and $\bar{C}measured$ is the mean of unadjusted pDR-1500 mass concentrations measured over the duration of the 38 locations. The pDR-1500 filter collected mass concentrations over an 8-h period. The percent difference was calculated using Equation (4) between the calculated PM sensor mass concentration and the filter-corrected pDR-1500 mass concentration measurements. The values of *A_i_* and *C* were the 1-min average mass concentrations for the PM sensors and pDR-1500, respectively. The data from the pDR-1000 used at the supersite and the pDR-1500 used for validation were both filter corrected, which is an important step to improve accuracy of the results.

## 3. Results and Discussion

### 3.1. Laboratory Selection of PM Sensors

**Figure 2:**
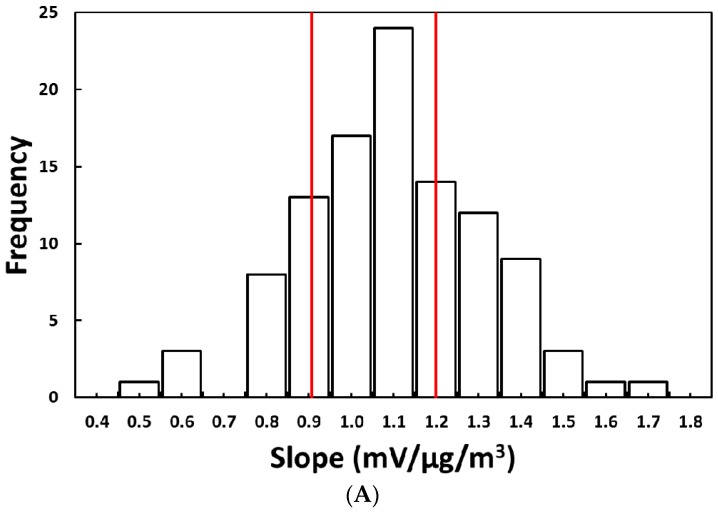

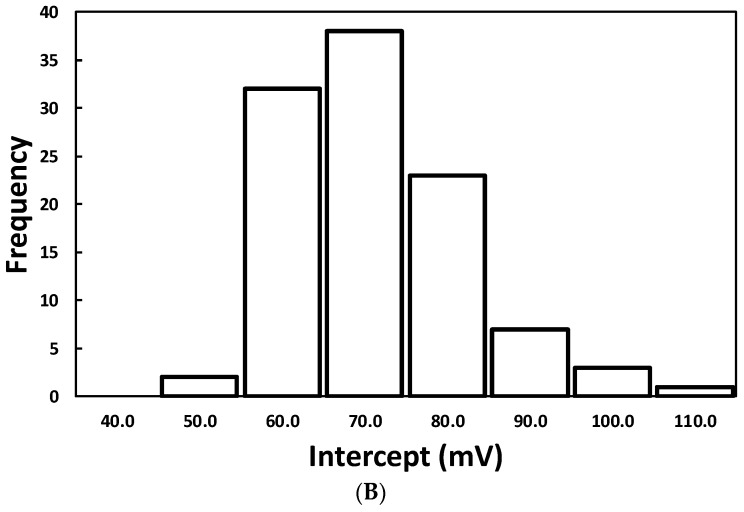
Frequency of (**A**) slopes (solid red lines represent a selection criterion of Z = ±14%) and (**B**) intercepts for the 100 PM sensors determined in laboratory experiments.

The slopes and intercepts of the 100 PM sensors varied substantially, with slopes ranging from 0.48 to 1.7 mV/µg/m^3^ and intercepts from 47 to 104 mV (Figure 2). Table 1 summarizes the slopes for the three PM sensors used in all three experiments. We found that the sensor responses were similar in the three experiments, where the coefficient of variation was less than 10%. The relative humidity, measured using the pDR-1500, was relatively constant, with a mean value of 29% and a 1.0% standard deviation. The chamber experiment was conducted in a controlled environment, and the low relative humidity is expected to have little impact on the Sharp GP sensor output [30]. Sousan, et al. [31] evaluated the homogeneity of the sampling zone, where the authors calculated the precision for four pDR-1500 sensors located at different positions for two aerosol types: salt and Arizona road dust. They found that the coefficient of variation was less than 10% for both aerosol types, indicating that the measurements were similar at different locations in the sampling zone.

The coefficient of determination compared to the reference instrument for the laboratory experiments was high for all sensors (*R*^2^ > 0.99). The fact that *R*^2^ is high means that the Sharp GP sensors and the pDR-1500 behave similarly. The finding that slopes and intercepts varied dramatically between sensors indicates that sensors require individual calibration, as suggested by Sousan, Koehler, Thomas, Park, Hillman, Halterman and Peters [23]. Calculated intercepts compared to values measured at zero concentration (mV) for 100 sensors are shown in Figure S1 in the Supplemental Information. The intercepts and the respective zero values (mV) are on or close to the 1:1 lines; therefore, we can assume that both values are equal. Thus, the laboratory intercepts or zero values can be used in the field to calculate mass concentrations. 

The selection criterion, based on Method 1, was Z = ±14% because it provided 50 PM sensors. The chosen sensors had an average slope of 1.07 mV/µg/m^3^, which was used to calculate mass concentration for all the sensors. The solid red lines in Figure 2A represent the ±14% criterion. The slopes for the 50 selected PM sensors ranged between 0.91 and 1.2 mV/µg/m^3^.

**Figure 3:**
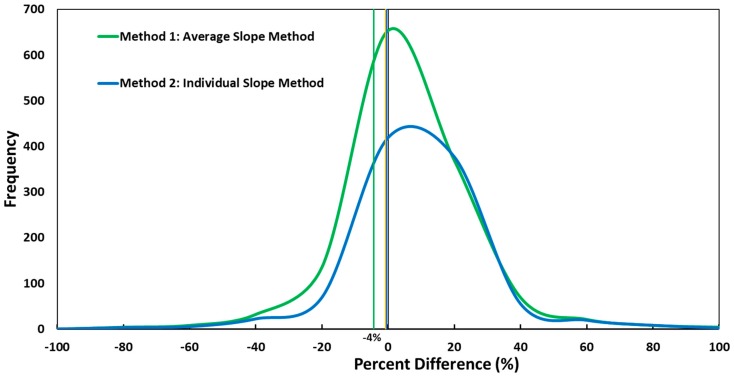
Frequency of percent differences to compare different calibration and selection methods for 1-min averages.

Figure 3 shows the frequency of percent differences for the mass concentration estimated by the PM sensors relative to the mass concentration from the pDR-1500 for the three selection methods at 1-min averages. The percent differences randomly vary around the zero percent value (ideal value). For Methods 1 and 2, the majority of the percent differences were −20% to 40%. The absolute mean percent difference for Method 1 was 13% compared to 12% for Method 2. Therefore, using an individual slope for each sensor is not recommended because it will require calibrating all the sensors for a minor improvement. We concluded that using Method 1, the Average Slope Method, was the best method to decrease percent differences around the zero value and requires field calibration of one or three PM sensors if precision is required.

The percent differences for all three methods exceeded ±10% NIOSH gas instrument criterion. However, the NIOSH criterion was established for comparing gas sensors with gas generation at stable pressure and temperature. Aerosol generation is less stable, difficult to control, and has an oscillatory behavior around steady-state concentrations that increases with higher concentrations.

**Figure 4:**
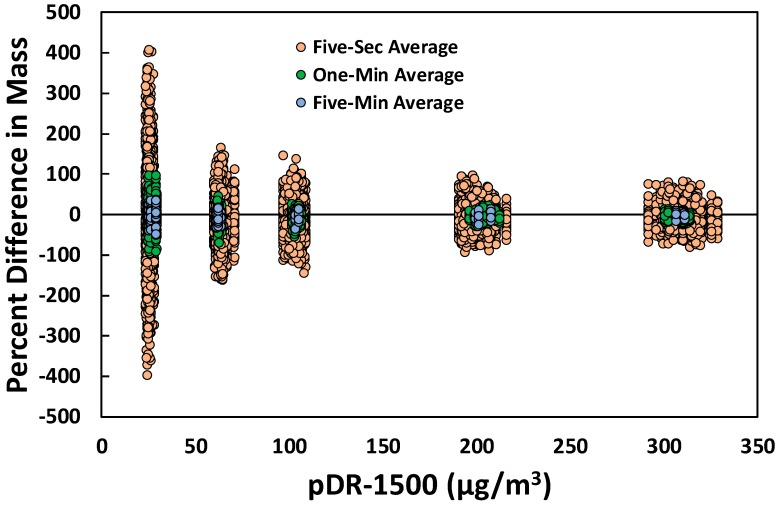
Percent difference between the calculated mass from the laboratory calibration of PM sensors and the pDR-1500 to compare different averaging times using Method 1, the Average Slope Method.

The percent differences, based on Method 1, for the 50 selected PM sensors compared to the pDR-1500 relative to the mass concentration, for three averaging times are shown in Figure 4. The 1-min data (green markers) shown in Figure 4 are the same percent differences for Method 1 shown in Figure 3. Similar to the previous section, the percent differences randomly vary around the zero percent value (ideal value). In addition, the variability in percent difference decreased with increasing concentration for all three methods. The fact that the percent differences decrease with higher concentrations was not surprising because the accuracy of the signal in mV increases with higher concentrations [32]. For the 5-s averaging time, the percent differences ranged from −397% to 407% for the lowest concentration and from −80% to 82% for the highest concentration. For the 5-min averaging time, the percent differences ranged from −47% to 34% for the low concentration and from −16% to 10% for the high concentration. The variability in percent differences also decreased considerably when the averaging time increased, especially for the low concentrations. For low concentrations, the variability in percent differences around the zero value were low compared to those for the 5-s averaging time. Therefore, increasing averaging times will help decrease percent differences around the zero value for low concentrations. Similarly, Zikova, Masiol, Chalupa, Rich, Ferro and Hopke [21] also concluded that calculating 1-h averages from 1-min data eliminates random noise in the measurements. 

We estimated LOD for the lowest concentration at 1-min averages to be 26 µg/m^3^ based on the sensors mean and standard deviation values of 26 and 8.62 µg/m^3^, respectively. The sensitivity of the sensors at the lowest concentration was 3.0 mV (standard deviation ±1.0), which provides an increase from 0 to 26 µg/m^3^.

**Figure 5:**
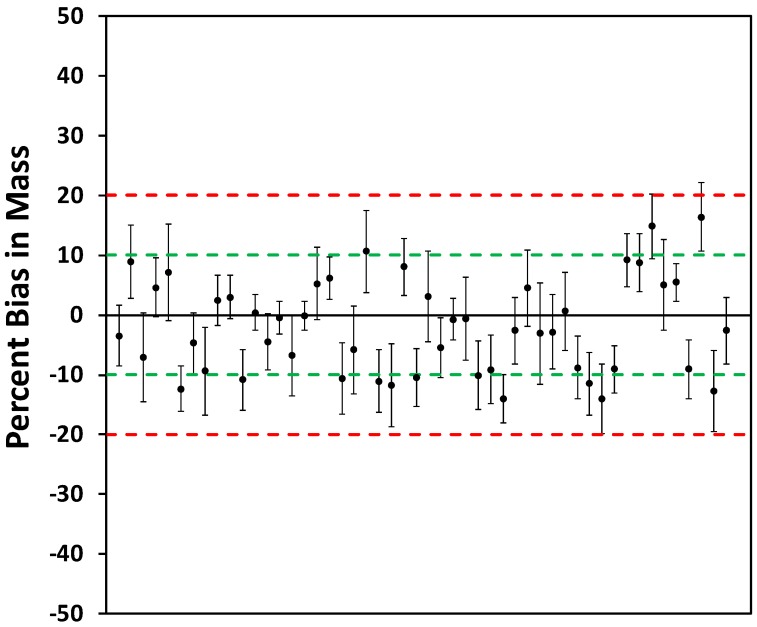
Bias value for the 50 selected PM sensors, based on Method 1, compared to the pDR-1500. The *y*-axis error bars represent the 90% confidence intervals. The dashed green and red lines represent 10% and 20% bias, respectively.

The bias values for the 50 selected PM sensors, based on Method 1, relative to the concentration measured with the pDR-1500 are shown in Figure 5. For each sensor, the bias was calculated based on 5-min averages for the five steady-state concentrations. The dashed green and red lines represent 10% and 20% bias, respectively. The mean bias values for all sensors were between −14% and 16%, with 38 PM sensors ≤ ±10%. The 90% upper and lower confidence interval varied between 2.4% for a −0.1% bias and 8.5% for a −3.1% bias, respectively. Only 12 PM sensors were outside the EPA bias criterion but were ≤±16%. Therefore, applying an average slope to 50 PM sensors provides bias values ≤ ±16%, where 41 out of 50 sensors were within EPA criterion of ≤±10%. The EPA 10% criterion was established for comparing PM sensors.

### 3.2. Application in the Field

The two-week measurement period for the three PM sensors and pDR-1000 at the supersite was interrupted. The pDR-1000 lost power after 12 days of data collection. In addition, one of the PM sensors lost wireless connection to the server and did not report measurements. Therefore, we calibrated the average mV values for two PM sensors with the filter-corrected pDR-1000 mass concentrations for 12 days. The average concentration for the unadjusted pDR-1000 at the supersite was 130 µg/m^3^ with a standard deviation of 35 µg/m^3^. The ratio of $\bar{G}measured/\bar{C}measured$ in Equation (8) was 2.85. The laboratory intercept values were used for each sensor. Therefore, zero values for each sensor were not subtracted before linear regression because the zero value only affects the intercept calculation and does not the affect the slope calculation. The slope and intercept were 0.29 mV/µg/m^3^ and 843 mV, respectively, based on the correlated (*R*^2^ = 0.9) average mV values for two PM sensors with the filter-corrected pDR-1000 mass concentration. The slope derived in the field, 0.29 mV/µg/m^3^, and the sensor-specific laboratory-derived intercepts were used to calculate the mass concentrations for all the PM sensors in the field. The relative humidity inside the manufacturing facility was controlled, and the pDR-1500 measured a low and relatively constant mean value of 25% with a 1.0% standard deviation.

**Figure 6:**
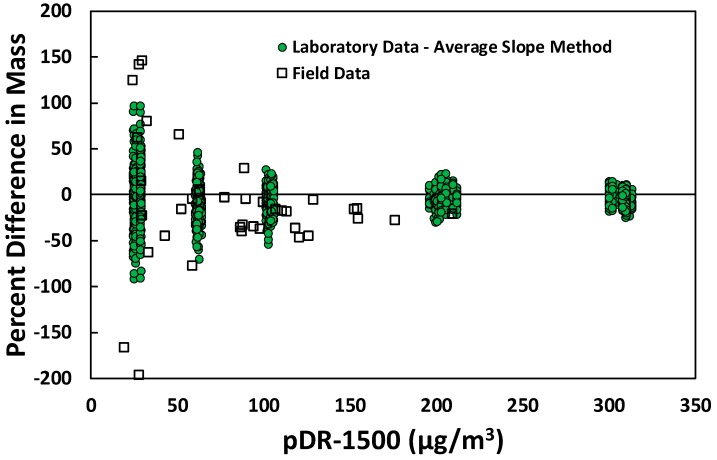
Comparison of percent difference observed in the laboratory to those observed in field tests based on 1-min averages.

The percent differences for 38 PM sensors relative to the unadjusted mass concentration from the pDR-1500 are shown in Figure 6. Another PM sensor was removed from our validation due to irregular mV values reported. The irregular values were caused by sensor defect and the sensor was replaced for further measurements. The two faulty sensors were not included in our field validation, and the sensors were replaced with backups for future assessment. We compared the 1-min average site-specific percent differences with the laboratory results from Method 1, the Average Slope Method. The average concentration for the unadjusted pDR-1500 while performing measurements at the 38 locations was 95 µg/m^3^ with a standard deviation of 53.79 µg/m^3^. The time required to complete the measurements at the 38 locations was 251 min. In order to compare the laboratory and field validation percent differences together, we used the unadjusted pDR-1500 measurements for the *x*-axis. The adjusted mass concentration in the field is much higher compared to mass concentrations generated in the laboratory. The ratio of $\bar{G}measured/\bar{C}measured$ in Equation (8) was 5.0. Therefore, the unadjusted pDR mass concentration was used to show both settings, laboratory and field, on the same *x*-axis. The percent differences, for field settings, were calculated based on the filter-corrected pDR-1500 mass concentration. The field percent differences (square markers) followed the same behavior as the laboratory values (circle markers), where the percent differences decrease with higher concentrations. The percent differences for 33 PM sensors were within the same range of the laboratory values (±100%). Five of the field PM sensors exceeded ±100 percent difference. However, the five sensors were at low concentrations, which is at the lower end of the calibration equation. Performing calibration for concentrations below 50 µg/m^3^ and using a separate regression equation might decrease the percent differences for these points. However, the field measurements are calculated at a 1-min average time resolution that increases the measurement noise. Performing 5-min collocated measurements should reduce measurement noise, but this would require five times the amount of time to perform measurements for all the sensors in the field. 

## 4. Conclusions

We proposed an Average Slope Method that allows selection of sensors that provide a similar response once calibrated within laboratory or field settings. The calculated percent difference range using an average slope was similar compared to the percent difference range calculated using an individual slope. We demonstrate that the average of multiple measurements over large time frequencies decreases the random noise dramatically, especially for lower concentrations. Averaging all the measurements for each sensor over a 25-min period provided bias values within ±16% for all the sensors. A field test suggested that a response derived on-site from only two sensors could be used to calculate mass for all the sensors chosen in the laboratory based on Method 1 with reasonable accuracy. The field calculated percent differences based on 1-min collocated averages matched the laboratory calculated percent differences for the majority of the sensors. We recommend contacting the manufacturer and requesting a batch of Sharp GP sensors that have a similar response. However, to our knowledge, we are not aware that this option is available.

## Figures and Tables

**Table 1:** Slopes (mV/µg/m^3^) for the three sensors used in the three experiments with the mean, standard deviation, and the percent coefficient of variation (CV) for each sensor.

Sensor Number	Experiment 1	Experiment 2	Experiment 3	Mean	Standard Deviation	CV (%)
1	0.92	0.81	0.92	0.88	0.06	7
2	0.74	0.75	0.71	0.73	0.02	3
3	1.07	1.05	1.04	1.05	0.02	1

## Supplementary Materials

The following are available online at http://www.mdpi.com/1424-8220/18/9/3008/s1, Figure S1, Intercept (mv) compared to the value measured at zero concentration (mV) for 100 sensors. The *x*-axis error bars represent one standard deviation.
