# Supplementary material for: Sensor Selection to Improve Estimates of Particulate Matter Concentration from a Low-Cost Network
Source: Sensors (Basel). 2018 Sep 8;18(9):3008. doi: 10.3390/s18093008 (PMC6163282; doi:10.3390/s18093008)
Supplement: Supplementary file 1 [file sensors-18-03008-s001.pdf]

sensors

MDPI

Supplementary Information

Sensor Selection to Improve Estimates of Particulate Matter Concentration from a Low-Cost Network

Sinan Sousan [1,2,*] Alyson Gray [2], Christopher Zuidema [3], Larissa Stebounova [2], Geb Thomas [4], Kirsten Koehler [3] and Thomas Peters [2]

[1] Department of Public Health, East Carolina University, Greenville, NC 27834, USA;
[2] Department of Occupational and Environmental Health, University of Iowa, Iowa City, IA 52242, USA; alyson-gray@uiowa.edu (A.G.); larissa-stebounova@uiowa.edu (L.S.); thomas-m-peters@uiowa.edu (T.P.)
[3] Department of Environmental Health and Engineering, Johns Hopkins Bloomberg School of Public Health, Baltimore, MD 21205, USA; czuidema@jhu.edu (C.Z.); kkoehle1@jhu.edu (K.K.)
[4] Department of Industrial and Systems Engineering, University of Iowa, Iowa City, IA 52242, USA; geb-thomas@uiowa.edu
* Correspondence: sousans18@ecu.edu

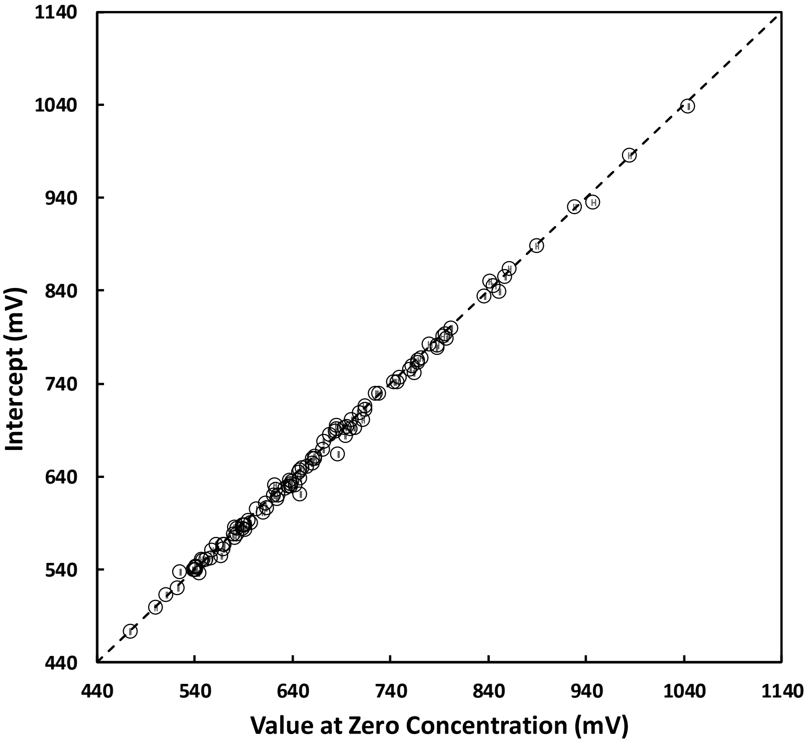

Figure S1. Intercept (mv) compared to the value measured at zero concentration (mV) for 100 sensors. The x-axis error bars represent one standard deviation.
